# Current and Future Approaches to Classify VUSs in LGMD-Related Genes

**DOI:** 10.3390/genes13020382

**Published:** 2022-02-19

**Authors:** Chengcheng Li, Gabe Haller, Conrad C. Weihl

**Affiliations:** 1 Department of Neurology, Washington University School of Medicine, Saint Louis, MO 63110, USA; chengchengl@wustl.edu (C.L.); ghaller@wustl.edu (G.H.); 2 Department of Neurological Surgery, Washington University School of Medicine, Saint Louis, MO 63110, USA; 3 Department of Genetics, Washington University School of Medicine, Saint Louis, MO 63110, USA

**Keywords:** LGMD, sarcoglycan, high-throughput screening, deep mutational scanning

## Abstract

Next-generation sequencing (NGS) has revealed large numbers of genetic variants in LGMD-related genes, with most of them classified as variants of uncertain significance (VUSs). VUSs are genetic changes with unknown pathological impact and present a major challenge in genetic test interpretation and disease diagnosis. Understanding the phenotypic consequences of VUSs can provide clinical guidance regarding LGMD risk and therapy. In this review, we provide a brief overview of the subtypes of LGMD, disease diagnosis, current classification systems for investigating VUSs, and a potential deep mutational scanning approach to classify VUSs in LGMD-related genes.

## 1. Introduction

Limb-girdle muscular dystrophies (LGMDs) are the fourth most common form of genetic muscle disease. They are defined by progressive weakness that predominantly affects the pelvic and shoulder girdle muscles, with age of onset ranging from early childhood to late adulthood. Based on their inheritance patterns, LGMDs are further subdivided into two main groups: autosomal dominant forms or LGMD1/LGMD-D, and autosomal recessive forms or LGMD2/LGMD-R [1]. To date, more than 30 genes associated with different subtypes of LGMD have been identified, with most of them being LGMD2. Compared to LGMD1, the LGMD2 subtype occurs much more frequently and is more common in childhood but it widely varies between subtypes and among different affected individuals. The genetic causes of these highly heterogeneous disorders have been widely investigated over the last sixty years, and LGMD is currently understood to be caused by gene mutations that result in abnormal proteins throughout muscle cells including in the extracellular matrix, sarcolemma, cytosolic contents, and nuclei [2,3].

Like other inherited disorders, diagnosis of LGMD begins with a detailed medical and family history followed by a physical examination. Ancillary testing is helpful and includes laboratory tests such as creatine kinase level determination, muscle magnetic resonance imaging, or electrodiagnostics. Final diagnosis often requires muscle biopsy processed via histochemistry and immunohistochemistry. However, due to large phenotypic heterogeneity and overlap across the LGMD subtypes, genetic testing is required for accurate determination of the LGMD genetic subtype. In some LGMD cases, Sanger sequencing is still considered standard and is the first diagnostic test performed, especially for known familial variant testing. For some patients, if protein-based assays and targeted Sanger sequencing are unable to identify the genetic causes, next-generation sequencing (NGS) is particularly useful. Recently, panel-based NGS has become widespread in the field of disease diagnosis, prediction, and risk assessment, showing high efficiency. Therefore, NGS can greatly help improve the diagnostic success rate of skeletal muscle disorders including LGMDs (reviewed by [4]). For example, whole-exome sequencing (WES, one strategy of NGS) followed by targeted analysis was reported to identify disease-causing mutations that were previously missed using incomplete Sanger sequencing [5], increasing the diagnostic success rate to 45% in a cohort of patients with LGMDs in Australia [6]. In contrast, high-throughput DNA sequencing technology can result in a large number of identified variants, with the majority of them considered variants of uncertain significance (VUSs), i.e., missense and intronic variants or in-frame insertions and deletions that have an unknown or unclear impact on protein function; therefore, their clinical significance is unknown. Nallamilli et al. [7] conducted NGS-based gene-panel testing on a cohort of 4656 patients from the United States with clinically suspected LGMDs, then diagnostic variants were interpreted. As high as 72% of the variants were classified as VUSs and only 26% of the variants were classified as pathogenic or likely pathogenic variants, according to the American College of Medical Genetics and Genomics (ACMG) criteria. The results from ClinVar, a freely available public archive of human genomic variants and interpretations of their relationship to diseases and other conditions [8], showed over 30,000 identified variants in established LGMD genes. Approximately 90% of the reported variants are single nucleotide substitutions, with half of them defined as VUSs (Table 1).

## 2. Current Guidelines and Methods for Variant Classification

The large number of genetic variants identified by widespread NGS of LGMD genes has created a strong demand for accurate classification of these variants that provides necessary information to make an informed clinical decision for early diagnosis or personalized treatment. Variant classification, the process of determining if a DNA variant causes disease, is currently based on the ACMG guidelines [9]. The latest revision recommends classifying variants into five categories based on criteria using evidence from literature reports, population frequency, mutation databases, computational data, and functional assays. Available databases used to annotate common variants in LGMD-related genes include the gnomAD, ClinVar, Leiden Open Variation Database (LOVD), and Human Gene Mutation Database [10]. Despite the comprehensive criteria provided by the ACMG, the process of variant classification is still changing and only a small portion of variants can be confidently predicted based on evidence. The majority of variants fail to be classified as either pathogenic or benign due to insufficient evidence or conflicting data. In these situations, the variants are often reported as VUSs.

VUSs represent an ongoing challenge to the interpretation process, as these variants are more often novel with little to no published data to support classification. Current methods to refine the pathogenicity of VUSs fall into two categories.

### 2.1. Co-Segregation and Biochemical Assays or Single-Variant Functional Assays

Classical approaches for interpreting variants require genotype–phenotype association studies in which large patient cohorts are needed to include enough patients with each variant to achieve statistical significance. This strategy has clear limits in interpreting variants associated with rare diseases such as LGMDs because the variants are present in only a few individuals. Another strategy is to broadly share genetic variants with little to no clinical information with clinical geneticists and research communities with the hope of aggregating genetic data across cohorts. Because VUSs are regularly identified from genome-wide testing, they can more easily be resolved through access to phenotype data from other individuals harboring the same variants observed elsewhere [11]. The main goal of global variant sharing is to enable robust diagnoses to be made as quickly as possible through collaborating and sharing detailed case-level information. A successful example is DECIPHER, a web-based international resource that aims to share and compare genomic and phenotypic data from patients with developmental disorders [12]. The DECIPHER database contains variant and phenotype information of tens of thousands of patients from more than 30 countries. Identification of additional patients worldwide who share common variants and phenotypic and clinical features aids the clinical interpretation of VUSs. To date, no global data-sharing resource has been developed specifically for LGMDs, but there are available resources such as the ClinVar which is funded by the NIH and was established as a database that provides a mechanism to upload and share phenotypic and clinical information on variants across the human genome [13,14]. Unfortunately, such data-sharing efforts might not greatly benefit the classification of variants in LGMDs, as many variants are only ever seen in a small number of individuals. A final strategy is to develop functional assessments, in vitro or in vivo, to evaluate the phenotypic consequences of gene variants, providing one of the strongest types of evidence recommended by the ACMG guideline for variant classification. For LGMDs, functional assays have traditionally been applied to each VUS as it is encountered in an individual. Due to its physiological and genetic similarities to humans, the mouse is the most commonly used model [15] to understand how specific variants identified in patients can influence the function of specific tissues in LGMDs. Recently, the zebrafish has become a useful vertebrate genetic model for human pathogenetic studies [16,17,18] due to low cost and relatively short generation time. Even though a combination of functional analysis and genetic testing has been used to elucidate some pathogenic mechanisms in LGMDs, the majority of LGMD variants remain uninterpretable or have conflicting interpretations. Individual assays have not generally been performed for rare clinical missense variants, and if they were, it was only after the discovery of the variant. In addition, these traditional assays are both time- and resource-intensive. Thus, this approach is clearly limited in scale to tens or hundreds of variants.

### 2.2. Computational/In Silico Prediction Tools

In order to rapidly predict variant effects and serve as a basis for clinical decision making, many computational or in silico approaches have been developed that rapidly analyze all possible variants of a gene of interest by predicting their effects at both the nucleotide and amino acid level. In silico refers to computational tools in chemistry, biology, and pharmacology that determine the effect of variants on primary and alternative gene transcripts and other genomic elements as well as the potential impact of variants on protein structure, activity, post-translational modification, and protein–protein interaction. Several methods have successfully been applied to human disease modeling, drug discovery, and variant interpretation. Certain types of variants detected by genetic testing using methods such as short insertions, deletions, and truncating mutations are more easily classified as pathogenic by in silico prediction tools, because their effect on protein structure and function is more evident.

Missense variants in protein-coding regions, which comprise 45% of variants implicated in disease [19], are more difficult to classify. Until now, in silico prediction algorithms such as PolyPhen2 [20], SIFT [21], and Mutation Taster [22] have often been used to predict pathogenesis of the missense variants in LGMDs. In most situations, more than one of the above prediction algorithms is used to predict the functional consequences of variants and potentially explain the pathogenic causes of LGMDs [23,24,25]. Furthermore, to take the results of many prediction tools into account, less biased methods such as CADD [26] and REVEL [27] were developed and became preferred when assessing variants. Beyond primary prediction of protein structure and function, pathogenicity prediction tools used for protein networks and pathways [28] and phosphorylation [29] also reported on LGMD genes. To date, prediction tools are mainly used on variants in coding regions or variants affecting splicing, and newer tools are beginning to address additional non-coding sequences [26].

Currently, for variant prediction of LGMD genes, computational prediction is the only approach that can provide evidence at a large scale. Unfortunately, this high-throughput approach poses certain limitations. It can identify only a small fraction of pathogenic variants with high confidence because none of these tools reach an accuracy above 90% [30]. A recent evaluation of predictor performance on 22 human disease genes revealed that with a threshold detecting 80% of pathogenic variants, the false prediction rate was 36% [31]. The rate of false positive or negative results was even higher when using genomes from patients of African descent, which harbor the highest genetic diversity [32]. Thus, misclassified variants may be present in the scientific literature and variant databases, greatly interfering with the interpretation of diagnostic sequencing results. One study reclassified 176 DYSF variants in a large French series of dysferlinopathy patients and revealed changed pathogenicity for 17 variants [33]. To overcome the limitation of poor accuracy, using more than one in silico tool is recommended. A consensus prediction between the different tools was obtained by identifying predicted protein disruptions that were consistent among all the tools with the highest confidence. However, different computational prediction algorithms may produce conflicting information [34,35]. In this situation, in vivo or in vitro functional characterization is needed to validate the in silico models.

## 3. The Emergence of High-Throughput Functional Assays

To overcome the limitations of biochemical assays and computational predictions, an experimental approach to assess thousands of variants simultaneously is needed. Deep mutation scanning (DMS), a technology collectively pioneered by Fowler et al. [36], Ernst et al. [37], and Hietpas et al. [38], among others, can be used to measure the functional consequences of variants on a massive scale, especially those in coding regions. This high-throughput approach has been widely used to assess the effects of variants identified in promoters [39], enhancers [40], splice sites [41,42], and UTRs [43,44], collectively called multiplex assays of variant effect (MAVE) [45]. In DMS, a pooled library is constructed that includes all possible single nucleotide or amino acid variants in a specific gene. There are a variety of methods for making mutagenesis libraries, including simple error-prone PCR [46] and our recently published approach using reversibly terminated inosine for more even representation [47]. The library of variants is then introduced into a model system where the genotype is linked to the phenotype. Model systems that have been used in DMS are typically bacteria, yeast, or cultured human cells, since mouse and zebrafish models used for single variant assays are not suitable for large-scale screening. After specific cells are selected based on protein function, high-throughput sequencing can be used to create sequence-function maps.

All instances of DMS share a similar process, but the type of functional assay is often specific to a protein and its function being tested, which can include the impact of variants on protein structure, catalytic or enzymatic activity, stability, ligand binding, protein interaction, or the expression of a fluorescent protein. The most frequently used functional assay is growth-based. This assay is usually performed in yeast or cell lines where the pathogenesis of a variant is coupled with the organism’s survival with or without a drug selection [48]. Phage display is another widely used assay to select clones with corresponding variants. Phage display systems display proteins on the surface of a phage, and the selection is made with the use of specific antibodies or ligands [49,50]. Selection based on fluorescence-activated cell sorting (FACS) is useful for investigating variants that affect enzyme activity, protein stability, or protein abundance [51,52,53], where the fluorescence intensity is proportional to the features of proteins. In addition, luciferase reporter assay was reported to evaluate the impact of variants on splicing [54] and variants within the non-coding regions [39]. Moreover, several groups combined the results from multiple DMS studies [53] or integrated DMS in computational prediction tools [55] to improve the accuracy of variant classification.

Deep mutational scanning approaches have been widely applied to cancer-risk genes. For example, with the use of DMS, more than 2000 missense variants in BRCA1 were measured [50], improving the diagnosis and understanding of disease risk. However, none of the LGMD-associated genes have been analyzed by this method. Because of its high throughput and accuracy, we think that this technology represents a viable strategy for overcoming the challenges associated with VUSs in LGMDs. In the remainder of this review, we propose a deep mutational scanning protocol using sarcoglycans as an example to discuss the feasibility of applying this technology on LGMDs.

## 4. Proposed Deep Mutational Scanning on Sarcoglycan Proteins

Sarcoglycans are a family of transmembrane proteins found within the dystrophin–glycoprotein complex (DGC). DGC is a large molecular complex that plays both a mechanical and signaling role between the intracellular cytoskeleton and the extracellular matrix around muscle cells [56,57]. Four sarcoglycans have been identified in skeletal and cardiac muscle: α-, β-, γ-, and δ-sarcoglycan transcribed from gene SGCA, SGCB, SGCG, and SGCD, respectively. These four sarcoglycans form a tetrameric subcomplex, which together stabilizes the sarcolemma and protects the muscle fibers from contraction-induced injury [58]. Additional ε- and ζ-sarcoglycans were found in smooth muscle, replacing α- and γ-sarcoglycan in the sarcoglycan complex [59,60].

**Figure 1:**
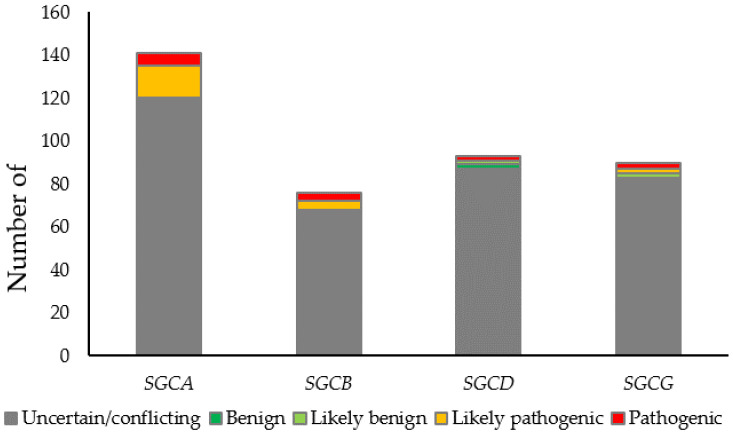
Number of missense variants identified in sarcoglycan genes. Variants were classified according to ACMG guidelines. The majority of identified variants are classified as VUSs or variants with conflicting interpretations.

**Figure 2:**
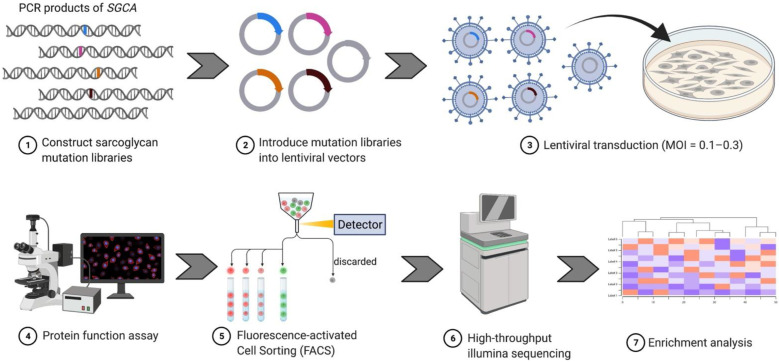
The workflow for high-throughput functional screen of SGCA. A comprehensive library containing every possible missense variant in the SGCA gene was delivered into a cell-based model system via lentiviral transduction at an MOI of 0.1–0.3. Non-permeabilized cells were then labeled by an antibody against an extracellular epitope of α-sarcoglycan and subjected to selection for membrane localization by FACS. DNA from selected cells was deeply sequenced to derive a functional score for each variant.

Sarcoglycans were first associated with LGMD in a report of a severe form of muscular dystrophy (SCARMD) affecting 93 children in Tunisia [61]. Affected children were characterized by muscle atrophy predominantly in the girdle and truncal muscles and showed markedly high creatine kinase activity between the ages 3 to 12 years. In 1992, chromosomal linkage analysis revealed that the defective gene associated with SCARMD was located on chromosome 13q12, later known as SGCG [62]. The same year, biopsies of four SCARMD patients showed deficiency in a 50 kd protein called adhalin [63] that was later renamed α-sarcoglycan. These observations support the complexity of this disease in which the loss of different types of sarcoglycan subunits leads to the same phenotype. We now know that the loss of any single sarcoglycan often leads to concomitant reduced or absent sarcolemmal expression of all four sarcoglycans [3,64,65]. However, conflicting data exist, suggesting that residual sarcoglycan may be present in some cases of limb-girdle muscular dystrophies. Mutations of any of the sarcoglycan complex subunits (α, β, δ, and γ) cause four distinct categories of limb-girdle muscular dystrophy: LGMD 2D, 2E, 2F, and 2C, respectively. Collectively called sarcoglycanopathies, they are one of the most common LGMDs, accounting for 5% to 10% of cases and >60% of severe cases. Phenotypically, progression of the disease ranges from very mild to very severe Duchenne-like muscular dystrophy [66,67,68]. In general, muscle biopsies from patients with sarcoglycanopathies show deficiency in one or more sarcoglycans, which makes the diagnosis challenging without clear and unambiguous genetic testing results. More complicatedly, some sarcoglycan mutations have been reported to be associated with respiratory failure and cardiomyopathy (reviewed by [69]). Thus, accurate diagnosis of sarcoglycanopathy relies on genetic testing. Variant interpretation becomes essential especially for missense variants for which pathogenesis is difficult to predict. Based on data from ClinVar, a total of 400 missense mutations have been identified in four sarcoglycan genes, with only 11% classified as pathogenic or benign (Figure 1). To the best of our knowledge, a high-throughput and reliable functional assay that enables more rapid and accurate interpretations of variants related to LGMDs is not yet developed. Here, we focused on missense and nonsense substitutions in the protein-coding region, as these variants are more likely to alter protein function [70]. We propose a DMS approach to classify all possible missense and nonsense mutations in sarcoglycan genes (SGCA as an example). The workflow is shown in Figure 2.

As the first step of designing a DMS protocol, mutational libraries were constructed to cover all possible single amino acid changes throughout the entire *SGCA* gene. This step was performed by cloning mutant oligonucleotide pools into a lentiviral vector. The libraries with thousands of different variants were then packaged into lentivirus, followed by expression in our cell-based model where the genotype is linked to phenotype. The multiplicity of infection (MOI) was controlled to be 0.1–0.3 so that each transduced cell would express only one *SGCA* variant. The design of the functional assay for mutant selection was based on the fact that α-sarcoglycan is a surface protein, and disease-causing variants often lead to premature degradation and deficient trafficking, resulting in the absence of sarcoglycan on the cell surface. In this case when non-permeabilized cells were labeled with fluorescent-conjugated sarcoglycan antibodies, cells harboring pathogenic variants were more likely to exhibit little to no surface sarcoglycan expression and show low to no fluorescent signal. In contrast, cells carrying benign variants were more likely to show high fluorescent signal. Fluorescently labeled cells were then sorted into different bins according to fluorescence intensity and further subjected to high-throughput sequencing to measure the frequency of variants across bins.

In our DMS approach, we constructed saturation mutant libraries for *SGCA* in which each amino acid was substituted with each of the other 19 common amino acids or a premature stop at every single position. Although some substitutions are unlikely to occur naturally, their inclusion could still provide us valuable insight. However, our libraries were limited to missense and nonsense *SGCA* variants. The functional impact of other variants such as frameshift, indels, splice variants, and variants in non-coding regions needs to be further evaluated. Pathogenic variants in *SGCA* resulted in the mislocalization of α-sarcoglycan to the cytoplasm but not the cell membrane, which allowed us to separate variants by fluorescence labeling assays. Variants associated with differing levels of α-sarcoglycan on the cell membrane could be separated by FACS, and they were all subsequently sequenced by NGS. Our functional assay is considered a well-established functional approach that can provide strong evidence in *SGCA* classification. The ACMG provides two sets of criteria for variant classification, including the strong evidence codes PS3 (well-established functional assays show deleterious effect) and BS3 (well-established functional assays show no deleterious effect). Our functional assay based on membrane localization of the α-sarcoglycan met criterion PS3 [71], and the variants that led to mislocalization of α-sarcoglycan were deleterious. However, our assay was not applicable to BS3 since the presence of membrane localization of the α-sarcoglycan is suggestive of functionality but may include some pathogenic variants. Protein membrane localization may not necessarily reflect normal protein function, especially for sarcoglycans that form a complicated tetrameric complex. Thus, proper assembly of the sarcoglycan complex may represent a better way to study sarcoglycan functions in the context of sarcoglycanopathies. Therefore, in our current project of classifying *SGCB* variants, we started evaluating the variant effect on complex assembly. Finally, preliminary data on *SGCA* variant classification show a bimodal distribution of variant types within our mutational library, indicating the presence of potential pathogenic and benign variants. However, interpreting the variants that fall between these two populations, such as variants that lead to a mild or impenetrant phenotype, is challenging. Thus, our current approach is biased toward severe pathogenic variants and leads to some variants not being resolved by DMS. To accurately annotate these variants, a secondary in vivo validation is needed.

## 5. Conclusions and Future Perspectives

Next-generation sequencing is steadily becoming more common in accurate disease diagnosis. Large-scale sequencing results in the identification of enormous numbers of genetic variants with unknown clinical significance. Resolving the functional consequence of variants is an ongoing challenge. As we described in this review, there are several approaches that are currently being used for VUS classification. However, they are limited by throughput and accuracy. A high-throughput screening approach that can functionally test the effects of thousands of variants at once has not yet been reported on LGMD genes. We proposed a DMS approach to scan all possible missense and nonsense mutations throughout the SGCA coding region, regardless of whether they were previously reported in a patient. A similar approach is being tested on other sarcoglycan genes to improve the classification of VUSs. Furthermore, to make our screening data easily accessible for research and clinical use, we publicly shared our data using the NIH sequencing reads archives (SRA) for raw data and the Gene Expression Omnibus (GEO) database for estimated functional scores. Our goal is to share data and interpretations for all tested variants via online portals with online user interfaces to enable rapid searches for specific variants and evidence that supports their pathogenicity (clinical data, in silico predictions, and functional scores from DMS). We think that our functional classification of VUSs supports immediate diagnostic interpretations of newly identified genetic variants and the application of this high-throughput approach for additional LGMD genes improves the diagnostic rate of LGMDs.

## Figures and Tables

**Table 1:** Number of identified variants and VUSs in genes related to LGMD in ClinVar.

Designation	Gene	Protein	# of Variants	# of VUSs
LGMD D1	*DNAJB6*	HSP40	346	116
LGMD D2	*TNPO3*	Transportin 3	323	136
LGMD D3	*HNRNPDL*	Heterogeneous nuclear ribonucleoprotein D-like protein	158	69
LGMD D4 and LGMD R1	*CAPN3*	Calpain3	1047	388
LGMD D5	*COL6A1*	Collagen 6α1	1219	440
	*COL6A2*	Collagen 6α2	1371	509
	*COL6A3*	Collagen 6α3	1972	957
LGMD R2	*DYSF*	Dysferlin	2129	687
LGMD R3	*SGCA*	α-sarcoglycan	422	140
LGMD R4	*SGCB*	β-sarcoglycan	327	148
LGMD R5	*SGCG*	γ-sarcoglycan	410	142
LGMD R6	*SGCD*	δ-sarcoglycan	510	257
LGMD R7	*TCAP*	Telethonin	200	106
LGMD R8	*TRIM 32*	Tripartite motif containing 32	410	249
LGMD R9	*FKRP*	Fukutin-related protein	573	256
LGMD R10	*TTN*	Titin	17,986	7315
LGMD R11	*POMT1*	Protein-O-mannosyl transferase1	609	241
LGMD R12	*ANO5*	Anoctamin 5	786	395
LGMD R13	*FKTN*	Fukutin	599	263
LGMD R14	*POMT2*	Protein-O-mannosyl transferase 2	604	297
LGMD R15	*POMGnT1*	Protein-O-linked mannose β 1,2 Nacetylglucosaminyl transferase 1	662	260
LGMD R16	*DAG1*	Dystroglycan	376	213
LGMD R17	*PLEC*	Plectin	3355	1677
LGMD R18	*TRAPPC11*	Transport protein particle complex 11	572	205
LGMD R19	*GMPPB*	GDP-mannose pyrophosphorylase B	203	80
LGMD R20	*ISPD*	Isoprenoid synthase domain	239	129
LGMD R21	*POGLUT1*	Protein O-glucosyltransferase 1	81	14
LGMD R22	*COL6A2*	Collagen 6α2	1371	509
LGMD R23	*LAMA2*	Laminin α2	2330	756
LGMD R24	*POMGnT2*	Protein-O-linked mannose β 1,2 Nacetylglucosaminyl transferase 2	267	143
LGMD R25	*BVES*	Blood vessel epicardial substance	66	5
LGMD R26	*POPDC3*	Popeye domain containing 3	29	2
LGMD R27	*JAG2*	Jagged canonical Notch ligand 2	98	13

Data available on ClinVar. Website: https://www.ncbi.nlm.nih.gov/clinvar/ (accessed on 6 January 2022).
